# Unlocking the *in vitro* anti-inflammatory and antidiabetic potential of *Polygonum maritimum*

**DOI:** 10.1080/13880209.2017.1301493

**Published:** 2017-03-17

**Authors:** Maria João Rodrigues, Luísa Custódio, Andreia Lopes, Marta Oliveira, Nuno R. Neng, José M. F. Nogueira, Alice Martins, Amélia P. Rauter, João Varela, Luísa Barreira

**Affiliations:** a CCMAR, Centro de Ciências do Mar, Universidade do Algarve, Faro, Portugal;; b Centro de Química e Bioquímica, Departamento de Química e Bioquímica, Faculdade de Ciências, Universidade de Lisboa, Lisboa, Portugal

**Keywords:** Halophytes, antioxidant activity, oxidative stress, macrophages, nitric oxide, α-amylase, α-glucosidase

## Abstract

**Context:** Several *Polygonum* species (Polygonaceae) are used in traditional medicine in Asia, Europe and Africa to treat inflammation and diabetes.

**Objective:** Evaluate the *in vitro* antioxidant, anti-inflammatory and antidiabetic potential of methanol and dichloromethane extracts of leaves and roots of the halophyte *Polygonum maritimum* L.

**Material and methods:** Antioxidant activity was determined (up to 1 mg/mL) as radical-scavenging activity (RSA) of 2,2-diphenyl-1-picrylhydrazyl (DPPH), 2,2′-azino-bis(3-ethylbenzothiazoline-6-sulphonic acid) (ABTS), copper (CCA) and iron (ICA) chelating activities and iron reducing power (FRAP). NO production was measured in lipopolysaccharide (LPS)-stimulated macrophages for 24 h at concentrations up to 100 μg/mL and antidiabetic potential was assessed by α-amylase and α-glucosidase inhibition (up to 10 mg/mL) assays. The phytochemical composition of the extracts was determined by gas chromatography-mass spectrometry (GC-MS).

**Results:** The methanol leaf extract had the highest activity against DPPH***•*** (IC_50_ = 26 μg/mL) and ABTS***^+^•*** (IC_50_ = 140 μg/mL), FRAP (IC_50_ = 48 μg/mL) and CCA (IC_50_ = 770 μg/mL). Only the dichloromethane leaf extract (LDCM) showed anti-inflammatory activity (IC_50_ = 48 μg/mL). The methanol root (IC_50_ = 19 μg/mL) and leaf (IC_50_ = 29 μg/mL) extracts strongly inhibited baker’s yeast α-glucosidase, but LDCM had higher rat’s α-glucosidase inhibition (IC_50_ = 2527 μg/mL) than acarbose (IC_50_ = 4638 μg/mL). GC-MS analysis identified β-sitosterol, stigmasterol, 1-octacosanol and linolenic acid as possible molecules responsible for the observed bioactivities.

**Conclusions:** Our findings suggest *P. maritimum* as a source of high-value health promoting commodities for alleviating symptoms associated with oxidative and inflammatory diseases, including diabetes.

## Introduction

The genus *Polygonum* (Polygonaceae) includes more than 200 species worldwide, mainly in areas of temperate climate. Several *Polygonum* species are used in traditional medicine in China and Japan to treat health disorders such as dysentery, articular pain and inflammation (Takasaki et al. 2001; Kawai et al. 2006; Fan et al. 2011). Some species are also used in traditional medicine in Europe, Africa and Asia to treat diabetes (Soumyanath 2005; Bothon et al. 2013). In Europe, approximately 36 species of *Polygonum* can be found, including *P. maritimum* L., commonly known as sea knotgrass. Sea knotgrass is a perennial halophyte herb native from the sandy coasts of Europe, Mediterranean and Black Sea, Channel Islands, England and Belgium, occurring frequently throughout the Portuguese coast (Kilinc & Karaer 1995; Caçador et al. 2013). *Polygonum maritimum* has described antioxidant and antimicrobial activities (El-Haci et al. 2013), and contains bioactive molecules such as polygonocinol, (+)-8-hydroxycalamene, octacosyl, triacontyl ferulate, arylpropane, quercetin, quercitrin, (+)-catechin, and sitosterol (Kazantzoglou et al. 2009).

Diabetes is an emerging health problem in western societies affecting more than 300 million people worldwide and is expected to be the 7th cause of death by 2030 (Mathers & Loncar 2006; Danaei et al. 2011). Type 2 diabetes mellitus (T2DM) is mainly associated with genetics and lifestyle and encompasses more than 90% of all diabetes cases globally (Mozaffarian et al. 2009). The major characteristic of T2DM is high blood glucose level, which is caused by congenital or acquired deficiency in secretion of insulin combined with decreased responsiveness to this hormone (WHO 1999; Yarchoan & Arnold 2014). The inhibition of carbohydrate-hydrolyzing enzymes, namely α-amylase and α-glucosidase, is thus an important strategy to manage hyperglycaemia linked to T2DM by decreasing the postprandial raise in blood glucose levels (Kwon et al. 2007). Acarbose, miglitol and voglibose are clinically used compounds that target α-amylase and α-glucosidase; however, they present several side effects such as abdominal distension, flatulence and meteorism (Bischoff & Flower 1985). In this sense, there has been a growing effort to search for novel natural compounds with antidiabetic properties and reduced side effects (Kwon et al. 2007).

Hyperglycaemia found in T2DM patients may also induce metabolic disturbances leading to the development of oxidative stress and chronic inflammation states that contribute to diabetes-associated complications, namely, cardiovascular, urological, neurological, kidney and eyes disorders (American Diabetes Association 2010; Vikram et al. 2014). Oxidative stress coupled with reduced antioxidant defences enhances damage caused by free radicals, such as reactive oxygen species (ROS), and contributes to disease progression (Sabu & Kuttan 2002; Maritim et al. 2003). In this context, natural antioxidants can be useful in the prevention and/or management of oxidative stress-related disorders, including diabetes (Ruhe & McDonald 2001; Fardoun 2007). ROS also contributes to the production of pro-inflammatory cytokines and chemokines and to insulin resistance (Akash et al. 2013; Muriach et al. 2014). The role of oxidative stress and chronic inflammation in the progression of T2DM thus opens new avenues in the search for novel and combined therapies comprising the prevention of oxidative and inflammatory states (Akash et al. 2013).

As stated before, several *Polygonum* species are used in traditional medicine to treat inflammation and diabetes. However, to the best of our knowledge, there is no information regarding the anti-inflammatory and/or antidiabetic potential of the sea knotgrass. In this context, we report for the first time a comparative evaluation of the antioxidant and anti-inflammatory potential and inhibitory activity on key enzymes relevant for hyperglycaemia (α-amylase and α-glucosidase) of extracts of sea knotgrass leaves and roots. The phytochemical characterization of the extracts is also presented.

## Material and methods

### Chemicals, culture media and supplements

Sigma-Aldrich (Germany) supplied the 1,1-diphenyl-2-picrylhydrazyl (DPPH), 2,2′-azino-bis(3-ethylbenzothiazoline-6-sulphonic acid (ABTS) radicals, sodium nitrite, lipopolysaccharide (LPS) from *Escherichia coli*, sulfanilamide, *N*-(1-Naphthyl) ethylenediamine dihydrochloride (NED) and 3-(4,5-dimethylthiazol-2-yl)2,5-diphenyl tetrazolium bromide (MTT). Folin-Ciocalteau (F-C) phenol reagent and phosphoric acid were purchased from Merck (Germany). Lonza (Belgium) provided Roswell Park Memorial Institute (RMPI) 1640 medium, fetal bovine serum (FBS), l-glutamine and penicillin/streptomycin. Additional chemicals were acquired from VWR International (Belgium).

### Sample collection

Whole plants of *P. maritimum* were hand collected in Ludo, South of Portugal, in June 2013. The taxonomical classification was performed by the botanist Dr. Manuel J. Pinto from the National Museum of Natural History (University of Lisbon, Botanical Garden, Portugal) and a voucher specimen is kept in the herbarium of MarBiotech laboratory (MBH22). Plants were divided in roots and leaves, washed, oven dried for 3 days at 50 °C, powdered and stored at −20 °C.

### Preparation of the extracts

Dried samples were separately extracted with methanol and dichloromethane (1:40, w/v), overnight at room temperature (RT), under stirring. The extracts were filtered (Whatman no. 4), and evaporated to dryness at 40 °C in a rotary evaporator under reduced pressure (BUCHI R-210, Flawil, Switzerland). The dried extracts were dissolved in the corresponding solvent at the concentration of 10 mg/mL to be used in the chemical characterization assays, or in dimethyl sulfoxide (DMSO) to be used in the bioactivity assays. All samples were stored at −20 °C until needed.

### Gas chromatography and mass spectrometry (GC-MS) phytochemical analysis

The extracts (100 μL) were filtered (0.2 μm polytetrafluoroethylene membrane syringe filters), transferred to a glass vial and the solvent evaporated under a nitrogen stream. When dried, 50 μL of the derivatization reagent [*N*-methyl-N-(trimethylsilyl) trifluoroacetamide; MSTFA] was added. With the vial capped, the extracts were vortexed and heated for 20 min in a dry block heater at 40 °C (Pereira et al. 2012).

The GC/MS analyses were performed on an Agilent 6890 series gas chromatograph equipped with an Agilent 7683 automatic liquid sampler coupled to an Agilent 5973 N mass selective detector (Agilent Technologies, Little Falls, DE). A programed temperature vaporization injector with a septumless sampling head (Gerstel, Mullheim a/d Ruhr, Germany) and a baffled liner was used, operating in the solvent vent mode with compressed air for inlet cooling. Large volume injection was performed (vent time, 0.30 min; flow, 50 mL/min; pressure, 0 psi; purge, 60 mL/min at 2 min), for which the inlet temperature was programed from 60 °C (0.4 min) to 300 °C (3 min isothermal) at a rate of 60 °C/min and subsequently decreased to 200 °C (held until end) at a rate of 50 °C/min. The injection volume and speed were set at 10 μL and 100 μL/min, respectively. GC analysis was performed on a Zebron ZB-5 (30 m × 0.25 mm I.D., 0.25 μm df; Phenomenex, USA) capillary column (5% phenyl, 95% polydimethylsiloxane), using helium as carrier gas maintained in a constant inlet pressure mode of 7.81 psi. The oven temperature was programed from 100 °C (1 min) at 20 °C/min to 250 °C, then at 10 °C/min to 300 °C and hold for 20 min. The transfer line, ion source and quadrupole analyzer temperatures were maintained at 280 °C, 230 °C and 150 °C, respectively and a solvent delay of 4 min was selected. In the full-scan mode, electron ionization mass spectra in the range 35-550 Da were recorded at 70 eV with an ionization current of 34.6 μA. Data recording and instrument control were performed by MSD ChemStation software (G1701CA; version C.00.00; Agilent Technologies).

### Radical-scavenging activity (RSA) on DPPH^•^

The RSA against DPPH was determined according to the method described by Custódio et al. (2015). Extracts (22 μL at concentrations ranging from 60 to 1000 μg/mL) were mixed with 200 μL of DPPH solution (120 μM, in methanol) in 96-well microplates and incubated in the dark for 30 min (RT). The absorbance was measured at 517 nm (Biotek Synergy 4) and results presented as half maximal inhibitory concentration (IC_50_, μg/mL). Butylated hydroxytoluene (BHT) was used as a positive control.

### RSA on ABTS^•^^+^

The RSA against ABTS radical was evaluated by the method described previously (Rodrigues et al. 2015). A stock solution of ABTS^•^^+^ (7.4 mM) was generated by reacting equal amounts of ABTS with potassium persulfate (2.6 mM) for 16 h in the dark at RT. The ABTS^•^^+^ solution was diluted with ethanol to obtain an absorbance of at least 0.7 at 734 nm (Biotek Synergy 4). Extracts (10 μL at concentrations from 60 to 1000 μg/mL) were mixed in 96-well microplates with 190 μL of ABTS^•^^+^ solution. After 6 min of incubation the absorbance was measured at 734 nm (Biotek Synergy 4). Results were expressed IC_50_ values (μg/mL). BHT was used as positive control.

### RSA on nitric oxide (NO^•^)

The NO^•^ scavenging activity was evaluated according to Rodrigues et al. (2015) on extracts at concentrations between 60 and 1000 μg/mL. Samples (50 μL) were mixed with 50 μL of 10 mM sodium nitroprusside in phosphate buffer (PBS) and incubated for 90 min at RT. After, 50 μL of Griess reagent (1% of sulfanilamide and 0.1% of naphthylethylenediamine in 2.5% HPO_3_) were added. The absorbance was read at 546 nm, and results were expressed as IC_50_ values (μg/mL). *N*_ω_-Nitro-l-arginine methyl ester hydrochloride (l-NAME) was used as standard.

### Copper (Cu^2+^) chelating activity (CCA)

The CCA was assessed using pyrocatechol violet as described previously (Rodrigues et al. 2015). The extracts (30 μL) were applied at concentrations from 60 to 1000 μg/mL and mixed with 200 μL of 50 mM Na acetate buffer (pH 6), 6 μL of pyrocatechol violet (4 mM) in the above buffer and 100 μL of CuSO_4_·5H_2_O (50 μg/mL, in water). The change in the colour of the solution was measured at 632 nm using a microplate reader (Biotek Synergy 4). Results were expressed as IC_50_ values (μg/mL). Ethylenediamine tetraacetic acid (EDTA) was used as a positive control.

### Iron (Fe^2+^) chelating activity (ICA)

The ICA chelating activity was determined by measuring the formation of the Fe^2+^ ferrozine complex (Megías et al. 2009), according to Rodrigues et al. (2015). Extracts (30 μL at concentrations between 60 and 1000 μg/mL) were mixed in 96-well microplates with 200 μL of dH_2_O and 30 μL of a FeCl_2_ solution (0.1 mg/mL in water). After 30 min, 12.5 μL of ferrozine solution (40 mM in water) was added. The change in colour was measured in a microplate reader (Biotek Synergy 4) at 562 nm, and results were expressed as IC_50_ values (μg/mL). EDTA was used as standard.

### Ferric reducing antioxidant power (FRAP) assay

The ability of the extracts to reduce Fe^3+^ was assayed by the method described by Megías et al. (2009). Extracts (50 μL) were tested at concentrations ranging from 60 to 1000 μg/mL and mixed with distilled water (50 μL) and 1% potassium ferricyanide (50 μL). After an incubation of 20 min at 50 °C, 50 μL of 10% trichloroacetic acid (w/v) and ferric chloride solution (0.1%, w/v) were added and absorbance was measured at 700 nm. Increased absorbance of the reaction mixture indicates increased reducing power. Antioxidant activity was calculated relatively to the positive control (BHT; 1000 μg/mL), and expressed as IC_50_ values (μg/mL).

### Cell culture and cell viability

RAW 264.7 macrophages were maintained in RPMI culture medium supplemented with 10% heat-inactivated FBS, 1% l-glutamine (2 mM), and 1% penicillin (50 U/mL)/streptomycin (50 μg/mL), and were maintained at 37 °C in humidified atmosphere with 5% CO_2_. Exponentially growing cells were plated in 96-well tissue plates at a concentration of 1 × 10^4^ cells/well and incubated for 24 h. Extracts were then applied at different concentrations (3 to 100 μg/mL) for 24 h. Control cells were treated with DMSO at the highest concentration used in test wells (0.5%), and cell viability was determined by the MTT colorimetric assay (Mosmann 1983). Briefly, 2 h prior to the end of the incubation period 20 μL of MTT (5 mg/mL in PBS) were added to each well and further incubated at 37 °C. Then, 150 μL of DMSO was added to each well in order to dissolve the formazan crystals and absorbance was measured at 590 nm (Biotek Synergy 4).

### Measurement of NO production

The NO production was evaluated using RAW 264.7 macrophages as described by Rodrigues et al. (2014). Cells were plated at 2.5 × 10^5^ cells/mL in 96-well tissue plates and allowed to adhere overnight. Afterwards, they were treated with nontoxic concentrations of the extracts, i.e., those that allowed cellular viability higher than 80%, in serum- and phenol-free culture medium, containing 100 ng/mL of LPS, for 24 h (Nishishiro et al. 2005). NO production was assessed using the Griess assay (Miranda et al. 2001). A calibration curve was prepared with different concentrations of sodium nitrite (1.5–100 μM). Results were expressed as percentage (%) of NO production, relative to a control containing DMSO (0.5%, v/v), and as IC_50_ values (μg/mL).

### α-Amylase inhibitory activity

The α-amylase inhibitory activity was determined by the method described by Xiao et al. (2006). Samples (40 μL at concentrations ranging from 1000 to 10,000 μg/mL) were mixed in 96-well microplates with 40 μL of amylase solution (100 U/mL in 0.1 M sodium phosphate buffer, pH 7.0) and 40 μL of 0.1% starch solution (diluted in the previous buffer). After 10 min at 37 °C, 20 μL of 1 M hydrochloric acid (HCl) and 100 μL of iodide solution (5 mM iodine (I_2_) + 5 mM potassium iodide (KI), in distilled water) were added and the absorbance was measured at 580 nm. Results were expressed as IC_50_ values (μg/mL). Acarbose was used as the standard at concentrations between 250 and 10,000 μg/mL.

### Baker’s yeast α-glucosidase inhibitory activity

Microbial (*Saccharomyces cerevisiae*) α-glucosidase inhibitory activity was determined according to the method described by Custódio et al. (2015). Samples (50 μL at concentrations ranging from 20 and 1000 μg/mL) were mixed with 100 μL of enzyme solution (1.0 U/mL, in 0.1 M sodium phosphate buffer, pH 7.0), and incubated for 10 min at 25 °C. Then, 50 μL of 5 mM *p*-nitrophenyl-α-d-glucopyranoside (NGP; diluted in the previous buffer) were added and incubated more 5 min at 25 °C. The absorbance was recorded at 405 nm using a microplate reader (Biotek Synergy 4) and results were expressed as IC_50_ values (μg/mL). Acarbose was used as positive control at concentrations from 250 to 10,000 μg/mL.

### Rat’s intestinal α-glucosidase inhibitory activity

Rat’s intestinal acetone powder was used as a crude enzyme extract as an example of enzyme of mammalian origin (Kwon et al. 2007). Rat’s intestinal acetone powder (250 mg) were mixed with 10 mL of 0.1 M sodium phosphate buffer (pH 7.0) and centrifuged at 5000 × *g* for 20 min at 4 °C. The supernatant (100 μL) was mixed with the extracts (50 μL at concentrations between 500 and 10,000 μg/mL), and incubated for 10 min at 25 °C. Then, 50 μL of 5 mM *p*-nitrophenyl-α-d-glucopyranoside (NGP; diluted in the previous buffer) was added and the mixture was incubated for 30 min at 37 °C. The absorbance was read at 405 nm using a microplate reader (Biotek Synergy 4), and results were expressed as IC_50_ values (μg/mL). Acarbose was used as positive control at concentrations from 250 to 10,000 μg/mL.

### Statistical analysis

Results were expressed as mean ± standard error of the mean (SEM), and experiments were carried out at least in triplicate. Significant differences were assessed by analysis of variance (ANOVA) followed by Duncan’s New Multiple Range Test, or by Kruskal–Wallis test when parametricity of data did not prevail. SPSS statistical package for Windows (release 15.0, SPSS Inc.) was used. The IC_50_ values were calculated by sigmoidal fitting of the data in the GraphPad Prism v. 5.0 software.

## Results

### Phytochemical analysis

**Figure 1.**
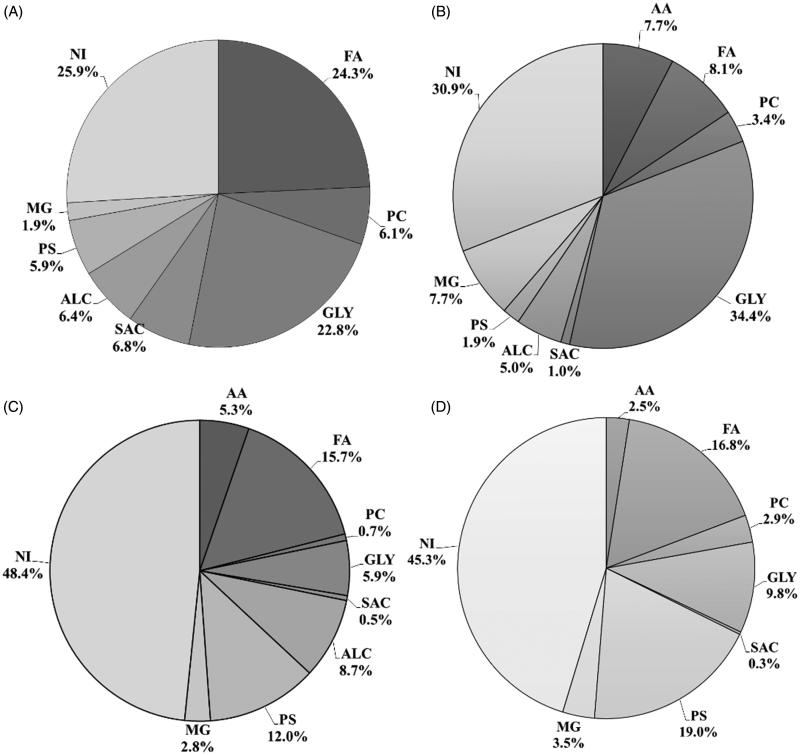
Main chemical compound classes identified by GC/MS in the dichloromethane and methanol extracts of roots and leaves of *P. maritimum*. (A) Methanol leaf extract; (B) Methanol root extract; (C) Dichloromethane leaf extract; and (D) Dichloromethane root extract. AA: alkanes and alkenes; FA: fatty acids; PC: phenolic compounds; GLY: acylglycerols; SAC: saccharides; ALC: alcohols; PS: phytosterols; MG: minor groups; NI: non-identified compounds.

In order to determine their chemical composition, extracts were analyzed by GC-MS (Figure 1 and Table 1). This analysis was able to identify a large number of compounds detected in the methanol extracts (70–75%). However, a large percentage of compounds detected in the dichloromethane extracts could not be identified (45–48%). In total, 51 compounds were identified belonging to different classes of biochemicals: alkanes and alkenes (AA), fatty acids (FA), phenolic compounds (PC), acylglycerols (GLY), saccharides (SAC), alcohols (ALC), phytosterols (PS) and minor groups (MG).

**Table 1.** Phytochemical composition of the methanol and dichloromethane extracts of *P. maritimum* leaves and roots, determined by GC/MS analysis.

				Relative abundance (%)			
				Methanol		Dichloromethane	
ID	RT (min)	Compound	Molecular formula	Leaves	Roots	Leaves	Roots
*Alkanes/Alkenes*							
**1**	5.61	2,6,11-Trimethyl dodecane	C_15_H_32_		0.67		
**2**	5.63	Phytane	C_20_H_42_			0.22	0.21
**3**	5.66	2,6,10,15-Tetramethyl heptadecane	C_17_H_36_		0.72		
**4**	6.75	Tetradecane	C_14_H_30_		0.97	0.32	0.40
**5**	8.55	Cetane	C_16_H_34_			0.16	0.18
**6**	8.63	2-Methyl octadecane	C_19_H_40_		0.78	0.26	
**7**	9.78	Octacosane	C_28_H_58_		0.77	0.37	0.70
**8**	9.90	3,11-Dimethyl nonacosane	C_31_H_64_		1.10		
**9**	11.57	Eicosane	C_20_H_42_		0.90		
**10**	12.39	Docosane	C_22_H_46_		1.78		1.01
**11**	12.40	Tricosane	C_23_H_48_			0.73	
**12**	14.10	Pentacosane	C_25_H_52_			0.59	
**13**	16.89	1-Nonadecene	C_19_H_38_			0.87	
**14**	17.75	Nonacosane	C_29_H_60_			1.80	
*Fatty acids*							
**15**	5.75	Pelargonic acid	C_12_H_18_O_2_	0.28			0.17
**16**	7.72	Lauric acid	C_12_H_24_O_2_	1.51		0.25	0.23
**17**	8.92	Myristic acid	C_14_H_28_O_2_	1.22		1.72	0.65
**18**	10.28	Palmitic acid	C_16_H_32_O_2_	6.40	1.46	4.49	6.53
**19**	11.08	Linoleic acid	C_18_H_32_O_2_	1.79	2.32	0.35	
**20**	11.18	Margaric acid	C_17_H_34_O_2_			0.14	0.20
**21**	11.75	Oleic acid	C_18_H_34_O_2_	4.13		2.32	3.21
**22**	11.85	Linolenic acid	C_18_H_30_O_2_			0.95	
**23**	11.94	Stearic acid	C_18_H_36_O_2_	6.71	4.27	1.83	1.38
**24**	13.59	Arachidic acid	C_20_H_40_O_2_	1.71		1.05	
**25**	15.32	Behenic acid	C_22_H_44_O_2_			1.35	2.64
**26**	17.10	Lignoceric acid	C_24_H_48_O_2_	0.53		1.29	1.78
*Phenols*							
**27**	5.09	Benzoic acid	C_7_H_7_O_2_	3.24	0.51		
**28**	6.94	Butylated hydroxytoluene	C_15_H_24_O	0.99	1.31	0.17	0.27
**29**	7.12	Vanillin	C_8_H_8_O_3_				0.68
**30**	7.70	Phloroglucinol	C_6_H_6_O_3_		0.92		
**31**	9.63	Methyl 3-(3,4-di-tert-butyl-4-hydroxyphenyl)propionate	C_18_H_28_O_3_	0.68	0.66		
**32**	9.69	Gallic acid	C_7_H_6_O_5_	1.15		0.54	1.97
*Acylglycerols*							
**33**	14.63	2-Monopalmitin	C_19_H_38_O_4_				1.28
**34**	14.89	1-Monopalmitin	C_19_H_38_O_4_	5.89	10.3	2.20	3.05
**35**	16.14	2-Monostearin	C_19_H_38_O_4_	15.4		0.34	
**36**	16.27	1-Monoolein	C_21_H_40_O_4_	0.35		0.47	
**37**	16.59	1-Monostearin	C_19_H_38_O_4_	1.08	24.1	2.87	5.44
*Saccharides*							
**38**	8.69	d-Fructose	C_6_H_7_O_6_	4.40	0.97	0.39	0.27
**39**	9.02	β-d-Glucofuranose	C_6_H_12_O_6_	0.85			
**40**	9.34	β-d-Glucopyranose	C_6_H_12_O_6_	1.05		0.14	
**41**	9.41	α-d-Glucopyranose	C_6_H_12_O_6_	0.45			
*Alcohols*							
**42**	4.12	Butane-1,3-diol	C_4_H_10_O_2_	0.62			
**43**	5.18	Glycerol	C_3_H_8_O_3_	5.20	5.02		
**44**	11.35	Phytol	C_20_H_40_O	0.62			
**45**	20.91	1-Octacosanol	C_28_H_58_O			8.72	
*Phytosterols*							
**46**	24.51	Stigmasterol	C_29_H_48_O			1.37	
**47**	25.88	β-Sitosterol	C_29_H_50_O	5.90	1.88	10.6	19.0
*Minor groups*							
**48**	5.25	Phosphoric acid	H_3_PO_4_	0.62	0.55	0.46	0.34
**49**	7.44	Diethyl phthalate	C_12_H_14_O_4_	0.35			
**50**	7.78	Thymine	C_5_H_6_N_2_O_2_		0.46		
**51**	13.11	Oleamide	C_18_H_35_NO	0.89	6.66	2.30	3.13

With similar contents, fatty acids (24.3%) and acylglycerols (22.8%) were the most represented categories in the methanol leaf extract (LM), in which 2-monostearin (**35**) was the major compound (15.4%). Palmitic acid (**18**), linolenic acid (**23**), 1-monopalmitin (**34**), glycerol (**43**), and β-sitosterol (**47**) were also detected at abundances higher than 5%. Acylglycerols were the most abundant constituents of the LM extract (34.4%), with 1-monostearin (**31**) being the most representative compound (24.0%), while 1-monopalmitin (**34**) and oleamide (**51**) represented 10.3 and 6.66% of the total identified components, respectively. In the dichloromethane leaf extract (LDCM), fatty acids represented 15.7% and phytosterols (PS) 12.0% of the total identified compounds. β-Sitosterol (**47**; 10.5%) and 1-octacosanol (**45**; 8.7%) were the major components identified. Similarly, the dichloromethane root extract (RDCM) had the highest content in phytosterols (PS; 19.0%) and fatty acids (FA; 16.7%), in which β-sitosterol (**47**, 19.0%) was the main component, followed by palmitic acid (**18**) and 1-monostearin (**37**) at 6.5 and 5.4%, respectively.

### Antioxidant activity

The *P. maritimum* LM and methanol root (RM) extracts showed high RSA for both DPPH and ABTS radicals, coupled with a strong capacity for reducing iron and chelating copper (Table 2). In the DPPH assay, the methanol extracts of both organs had similar IC_50_ values (roots: 27 μg/mL; leaves: 26 μg/mL), which were significantly lower than the one obtained with the positive control (BHT, 110 μg/mL; Table 2). Regarding the ABTS radical, both the RM and LM extracts had IC_50_ of 192 and 140 μg/mL, respectively, which were similar to that of BHT (140 μg/mL; Table 2). The RM and LM had also high ability to reduce iron with IC_50_ values of 64 and 48 μg/mL, respectively (Table 2). A moderate iron reduction was obtained upon addition of the RDCM (IC_50_ = 770 μg/mL) and LDCM (IC_50_ = 798 μg/mL) extracts. However, none of the extracts had the capacity for scavenging the NO radical or for chelating iron (Table 2).

**Table 2.** Radical scavenging activity (RSA) on DPPH, ABTS and NO radicals, metal chelating activity on iron (ICA) and copper (CCA) and ferric reducing activity (FRAP) activity of methanol (MeOH) and dichloromethane (DCM) extracts of roots and leaves of *P. maritimum.*

		RSA			Metal chelation/reduction		
Extract/Standard	Plant organ	DPPH	ABTS	NO	CCA	ICA	FRAP
MeOH	Leaves	26 ± 0.7^a^	140 ± 6^a^	n.a.	290 ± 10^b^	n.a.	48 ± 1.4^a^
	Roots	27 ± 0.0^a^	192 ± 5^b^	n.a.	446 ± 8^c^	n.a.	64 ± 3.8^a^
DCM	Leaves	n.a.	n.a.	n.a.	n.a.	n.a.	798 ± 36^b^
	Roots	n.a.	n.a.	n.a.	n.a.	n.a.	770 ± 53^b^
BHT*	–	110 ± 10^b^	140 ± 10^a^	–	–	–	–
l-NAME*	–	–	–	2500 ± 10	–	–	–
EDTA*	–	–	–	–	170 ± 10^a^	55.9 ± 3.7	–

Results are expressed as IC_50_ values (μg/mL). *Positive control; n.a.: not active. Values are means ± SEM of three separate experiments performed in triplicate (*n* = 9). In the same column, means labelled with different letters are significantly different by Duncan’s multiple range test (*p* < 0.05).

### Anti-inflammatory activity

To assess the *in vitro* anti-inflammatory activity, the effect of applying nontoxic concentrations of the extracts (i.e. yielding cell viability >80%) on the NO production by LPS-stimulated RAW 264.7 macrophage cells was determined. A significant reduction in cell viability was observed upon applying the LM extract at a concentration of 100 μg/mL to the cells. Loss of viability was also observed with the RDCM extract at 50 and 100 μg/mL (data not shown). Therefore, these concentrations were not used in the anti-inflammatory activity assessment.

**Figure 2.**
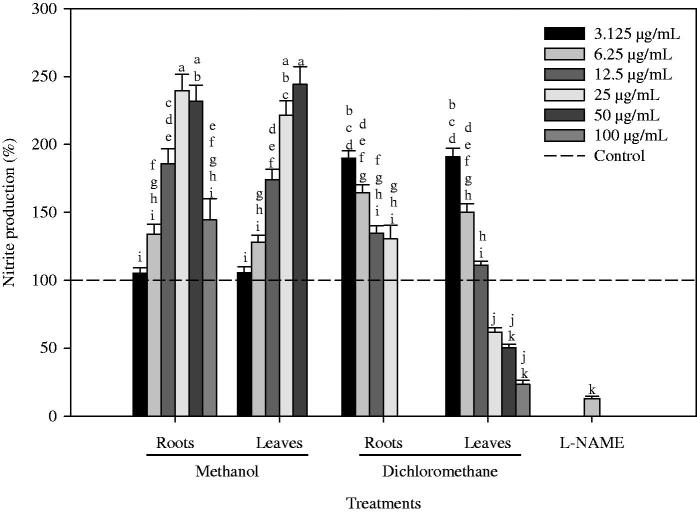
Effect of the application of dichloromethane and methanol extracts of roots and leaves of *P. maritimum* on NO production (%) by LPS-stimulated macrophages. Control cells were treated with culture medium supplemented with 0.5% DMSO and 100 ng/mL of LPS. l-NAME (positive control) was applied at the concentration of 100 μg/mL. Solid and errors bars represent the average and SEM, respectively (*n* = 9). Bars followed by different letters are significantly different according to the Duncan’s multiple ranges test (*p* < 0.05).

Exposure of RAW 264.7 cells to LPS at 100 ng/mL increased the nitrite concentration in the culture medium from a basal level of approximately 0.3 μM to around 13 μM (data not shown). This increase was significantly reduced in a dose-dependent manner by the treatment with LDCM, at concentrations ranging from 25 to 100 μg/mL, showing that the latter had an activity similar to the positive control (l-NAME) at the same concentration (Figure 2). However, this extract had an IC_50_ value of 48 μg/mL, higher than that of l-NAME (29.1 μg/mL; data not shown). Interestingly, incubating this cell line with the remaining extracts resulted in an increase in NO production when compared to the control (Figure 2). In particular, incubation with LM and RM extracts resulted in the most significant increases in NO production: 144% at the concentration of 50 μg/mL and 139% at 25 μg/mL, respectively.

### Antidiabetic activity

The inhibitory potential of extracts from *P. maritimum* was evaluated against α-amylase and baker’s yeast and rat’s α-glucosidases (Table 3). The methanol extracts had the highest capacity to inhibit the baker’s yeast α-glucosidase with IC_50_ values of 19 and 29 μg/mL for roots and leaves, respectively, which were significantly lower than that of acarbose (IC_50_ = 3144 μg/mL). Despite the fact that methanol extracts had no capacity to inhibit rat’s α-glucosidase, LDCM had the highest rat’s α-glucosidase inhibitory activity (IC_50_ = 2527 μg/mL), which was a significantly lower IC_50_ value than that of acarbose (4638 μg/mL; *p* < 0.05).

**Table 3.** Inhibitory activity of dichloromethane and methanol extracts of roots and leaves of *P. maritimum* on α-amylase, baker’s yeast α -glucosidase and rat’s intestinal α-glucosidase.

Extract/Standard	Organ	α-Amylase	Yeast α-glucosidase	Rat α-glucosidase
Methanol	Leaves	n.a.	29 ± 0.7^a^	n.a.
	Roots	n.a.	19 ± 0.5^a^	n.a.
Dichloromethane	Leaves	n.a.	585 ± 27^b^	2527 ± 37
	Roots	n.a.	626 ± 14^b^	>2500
Acarbose*		7797 ± 98	3144 ± 132^c^	4638 ± 438

Results are expressed as IC_50_ values (μg/mL). *Positive control; n.a.: not active. Values are means ± SEM of three separate experiments performed in triplicate (*n* = 9). In the same column, means labelled with different letters are significantly different by Duncan’s multiple range test (*p* < 0.05).

## Discussion

In order to confirm the use of *Polygonum* species in traditional medicine in the treatment of inflammation and diabetes, we assessed for the first time the phytochemical composition of *P. maritimum* and its *in vitro* anti-inflammatory and antidiabetic potential.

GC-MS analysis detected alkanes, alkenes and fatty acids as the major categories of compounds in *P. maritimum* extracts. These are lipophilic compounds widely distributed in plants as constituents of plant waxes and have been described in the halophilic Mediterranean *Suaeda vera* Forssk. ex J.F.Gmel. (Amaranthaceae), *Sarcocornia fruticosa* (L.) A.J.Scott (Amaranthaceae), and *Halimione portulacoides* (L.) Aellen (Amaranthaceae) (Grossi & Raphel 2003). Waxes usually have a protective role, for example, against microbial infections and avoiding excessive water losses and acute osmotic stress that halophilic species suffer while growing in drenched soils (Müller & Riederer 2005; Huang et al. 2011). Moreover, they are common constituents of essential oils of halophyte species, such as *Suaeda fructicosa* and *Limonium echioides* (L.) Mill. (Plumbaginaceae) (Saïdana et al. 2008). From the compounds detected in this study, only β-sitosterol (**47**) had been previously identified in *P. maritimum* also in dichloromethane extracts. Phytol (**44**), palmitic (**18**) and lauric (**16**) acids were also previously detected as components of essential oils of *Polygonum* species, namely *P. hydropiper* L. and *P. minus* Huds. (Miyazawa & Tamura 2007; Baharum et al. 2010).

Our findings showed that *P. maritimum* has a strong antioxidant activity comparable to that reported by other authors for similar extracts made from aerial parts of the same species (El-Haci et al. 2013) and *P. sachalinensis* F.Schmidt and *P. cuspidatum* Siebold & Zucc. (Pan et al. 2007; Fan et al. 2011). Moreover, IC_50_ values obtained with the RM and LM were significantly lower than or similar to the one obtained with the positive control (BHT) for DPPH and ABTS assays, respectively. The significantly higher antioxidant activity of these extracts can be related to the presence of some of the compounds identified in these extracts, namely, the phenolic compounds benzoic acid, BHT, vanillin and phloroglucinol, since these compounds are well described as strong antioxidants *in vitro* (Foti 2007; Dai & Mumper 2010). Phytol and linolenic acid have also been described as potent *in vitro* and *in vivo* antioxidants due to their hydroxyl group (Richard et al. 2008; Santos et al. 2013). Thus, their presence in the LM could also contribute to the high RSA and ICA of this extract. Interestingly, no significant differences were observed between the antioxidant activity of leaves and roots as opposed to that reported for other halophytes species, such as *Mesembryanthemum edule* L. (Aizoaceae)*, Limoniastrum monopetalum* (L.) Boiss. (Plumbaginaceae)*, Salsola kali* L. (Chenopodiaceae)*, Tamarix gallica* L. (Tamaricaceae) and *Limonium algarvense* Erben (Plumbaginaceae) (Ksouri et al. 2008; Falleh et al. 2012; Trabelsi et al. 2012; Rodrigues et al. 2015). This can be explained by the similar phytochemical profile of the methanol extracts of both organs (Figure 1 and Table 1), suggesting that the bioactive compound(s) are not organ-specific.

LPS is a cell wall endotoxin produced by Gram-negative bacteria that activates macrophages to produce inflammatory mediators such as NO (Martich et al. 1993), a radical associated with chronic inflammation (Kubes 2000; Joo et al. 2014). In this context, a decrease in NO production is used as an indicator of the potential for the extracts to reduce an inflammatory response (Joo et al. 2014; Rodrigues et al. 2014). On the other hand, an increase in NO production can indicate an immunostimulatory effect of the extracts, which is important in macrophage defence and protection against infection (Wink et al. 2011).

The anti-inflammatory effect of *P. maritimum* leaves may be attributed to the presence of compound(s) with potential anti-inflammatory properties, most likely β-sitosterol, stigmasterol, 1-octacosanol, oleamide as well as linolenic and oleic acids (Table 1). For instance, β-sitosterol, one of the major constituents of this extract, has anti-inflammatory properties through TNF-α inhibition (Loizou et al. 2010), and so do 1-octacosanol and oleic acid (Vassiliou et al. 2009; de Oliveira et al. 2012). In turn, linolenic acid and oleamide are able to reduce NO production and inducible nitric oxide synthase (iNOS) gene expression, through the inhibition of the NF-κB pathway (Ren & Chung 2007; Oh et al. 2010). Stigmasterol was also reported to inhibit the production of pro-inflammatory mediators associated with the same pathway (Gabay et al. 2010). The combination of all these compounds in the LDCM of *P. maritimum* are probably contributing to its NO inhibitory capacity, since they were solely identified in this extract, or in higher quantities (Figure 2). These findings are in accordance with several reports on the anti-inflammatory properties of *Polygonum* species (*P. lapathifolium L., P. cuspidatum* and *P. perfoliatum* L. (Takasaki et al. 2001; Kim et al. 2007; Fan et al. 2011; Lei et al. 2015).

NO has an important role in the immune system modulation, being one of the macrophage-mediated primary responses against pathogens such as fungi, helminthes, protozoa and bacteria (Wink et al. 2011). In this sense, the increased NO production suggests that both the LM and RM from *P. maritimum* may have immunostimulatory properties (Wink et al. 2011) most likely due to the presence of specific molecules in these extracts, namely, saturated fatty acids (stearic and palmitic acids), which are known inducers of the production of pro-inflammatory cytokines in macrophages (Valdearcos et al. 2012; Miao et al. 2015). In fact, immunostimulatory properties are reported in *P. multiflorum, P. minus* and *P. cuspidatum* (Chen et al. 2012; Veerasamy et al. 2014; Chueh et al. 2015).

Compounds with the capacity to inhibit carbohydrate-hydrolyzing enzymes like α-amylase and α-glucosidase can delay the digestion of carbohydrates, decreasing the postprandial increase of blood glucose level after a mixed carbohydrate meal, and therefore can be an important strategy in managing hyperglycaemia linked to T2DM (Krentz & Bailey 2005; Kwon et al. 2007; Bhandari & Ansari 2008).

It is noteworthy that methanol extracts were approximately 100-fold more active towards baker’s yeast α-glucosidase than acarbose and that the LDCM had 2-times more ability to inhibit rat’s α-glucosidase. High IC_50_ values for acarbose against this enzyme have also been reported by other authors (4823 μg/mL) (Gao et al. 2013). However, the methanol extracts had no capacity to inhibit rat´s α-glucosidase, which is a common feature of molecules with inhibitory capacity on α-glucosidase from microbial origin (Oki et al. 1999; Shai et al. 2011). Furthermore, a few *in vitro* studies have discussed the low capacity of acarbose to inhibit mammalian α-glucosidase compared to crude extracts, including aqueous ethanol extracts from *P. senegalensis* (Meisn.) Soják (Polygonaceae), which is used in folk medicine to treat T2DM (Shinde et al. 2008; Bothon et al. 2013). Those differences are usually attributed to additive or synergistic interactions of the compounds present in the extracts, resulting in a higher capacity to inhibit the mammalian α-glucosidase (Adisakwattana et al. 2012). The higher activity of the LDCM can be related with the presence of some particular compounds. For example, β-sitosterol was the main compound identified in this extract (Table 1) and was previously reported to possess strong hypoglycaemic activity through α-glucosidase inhibition (Ortiz-Andrade et al. 2007). The same properties were described for stigmasterol and linolenic acid, present only in this extract, as well as for oleic acid (Ortiz-Andrade et al. 2007; Lean Teik et al. 2013; Su et al. 2013). In fact, Su et al. (2013) reported that linolenic and oleic acids were more active than acarbose. Although none of the extracts achieved 50% of inhibitory activity in the α-amylase assay, our data suggest that *P. maritimum* may have potential as a source of antidiabetic molecules. This is in accordance with the antidiabetic activity found in several *Polygonum* species, such as *P. aviculare* L. (Polygonaceae)*, P. cuspidatum, P. multiflorum* and *P. senegalensis* (Soumyanath 2005; Bothon et al. 2013).

## Conclusions

The present study highlights for the first time the potential of the halophyte *P. maritimum* as a source of compounds with antioxidant, anti-inflammatory and antidiabetic activities. The methanol extracts had the highest antioxidant capacity, possibly due to the presence of benzoic acid, BHT, phloroglucinol, phytol and linolenic acid. The dichloromethane extracts from *P. maritimum* leaves had significant anti-inflammatory activity, most likely related to its main constituents identified as β-sitosterol, stigmasterol, 1-octacosanol, oleamide, linolenic and oleic acids. Moreover, its high α-glucosidase inhibitory activity may be related to the presence of β-sitosterol, stigmasterol, linolenic and oleic acid. Overall, our results indicate that *P. maritimum* extracts are endowed with compounds with potential to be used as a combined strategy to manage T2DM due to its anti-inflammatory, antioxidant and α-glucosidase inhibitory properties. These results could be the starting points to further explore *P. maritimum*, especially its leaves, as a source of value-added bioactive natural products. Nonetheless, isolation and identification of the molecules(s) responsible for the detected biological activities is already being pursued.
